# Putting human Tid-1 in context: an insight into its role in the cell and in different disease states

**DOI:** 10.1186/s12964-022-00912-5

**Published:** 2022-07-19

**Authors:** Sagarika Banerjee, Rupesh Chaturvedi, Anu Singh, Hemant R. Kushwaha

**Affiliations:** 1 School of Biotechnology, Jawaharlal Nehru University, New Delhi, India; 2 School of Biotechnology and Special Centre for Systems Medicine, Jawaharlal Nehru University, New Delhi, India

**Keywords:** hTid-1, Apoptosis, Mitochondria, Cancer, Tumorigenesis, Cardiac myopathies, Alzheimer’s disease, Parkinson’s disease

## Abstract

**Background:**

Tumorous imaginal disc 1 (hTid-1) or DnaJ homolog subfamily A member 3 (DNAJA3), is a part of the heat shock protein (Hsp) 40 family and is predominantly found to reside in the mitochondria. hTid-1 has two mRNA splicing variants, hTid-1S and hTid-1L of 40 and 43 kDa respectively in the cytosol which are later processed upon import into the mitochondrial matrix. hTid-1 protein is a part of the DnaJ family of proteins which are co-chaperones and specificity factors for DnaK proteins of the Hsp70 family, and bind to Hsp70, thereby activating its ATPase activity. hTid-1 has been found to be critical for a lot of important cellular processes such as proliferation, differentiation, growth, survival, senescence, apoptosis, and movement and plays key roles in the embryo and skeletal muscle development.

**Main body:**

hTid-1 participates in several protein–protein interactions in the cell, which mediate different processes such as proteasomal degradation and autophagy of the interacting protein partners. hTid-1 also functions as a co-chaperone and participates in interactions with several different viral oncoproteins. hTid-1 also plays a critical role in different human diseases such as different cancers, cardiomyopathies, and neurodegenerative disorders.

**Conclusion:**

This review article is the first of its kind presenting consolidated information on the research findings of hTid-1 to date. This review suggests that the current knowledge of the role of hTid-1 in disorders like cancers, cardiomyopathies, and neurodegenerative diseases can be correlated with the findings of its protein–protein interactions that can provide a deep insight into the pathways by which hTid-1 affects disease pathogenesis and it can be stated that hTid-1 may serve as an important therapeutic target for these disorders.

**Graphical Abstract:**

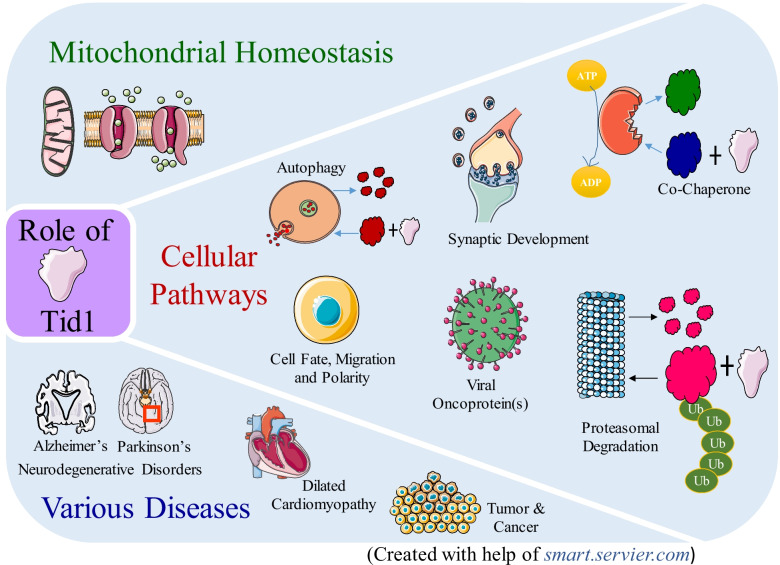

**Video Abstract**

**Supplementary Information:**

The online version contains supplementary material available at 10.1186/s12964-022-00912-5.

## Background

When cells are subjected to different environmental and physiological stresses such as exposure to cold, heat, UV light, etc., a natural defense mechanism of the cells is to dramatically amplify the expression of proteins called the Heat Shock Proteins (HSPs) [1–3]. Many of these proteins help in allaying the stress conditions in the cell by performing chaperone functions which help in ensuring the correct folding of new proteins and refolding of proteins damaged by cell stress, thereby allowing the cells to survive and function in these lethal conditions [4]. Two mitochondrial Heat Shock Proteins, the molecular chaperone HSP70, and its co-chaperone HSP40 are critically important in preventing the aggregation of misfolded proteins [5, 6]. An important member of the mitochondrial Hsp40 family, Tid-1, also called as DnaJ homolog subfamily A member 3 (DNAJA3) is a mammalian homolog of the *Drosophila* lethal tumorous imaginal disc Tid56 protein. The *Drosophila l (2) tid* gene, a tumor suppressor encodes the Tid56 protein, and null mutants of the protein result in a lethal phenotype wherein the imaginal discs of the fruit fly fail to differentiate and instead grow into lethal tumors [7]. hTid-1 has a highly conserved DnaJ domain with which it binds to Hsp70 to regulate the specificity and activity of their interacting substrate proteins [8, 9]. DnaJ domain-containing proteins function as co-chaperones to DnaK- like ATPases like Hsp70 to promote the folding, translocation, and degradation of interacting polypeptides [10–12].

**Fig. 1:**
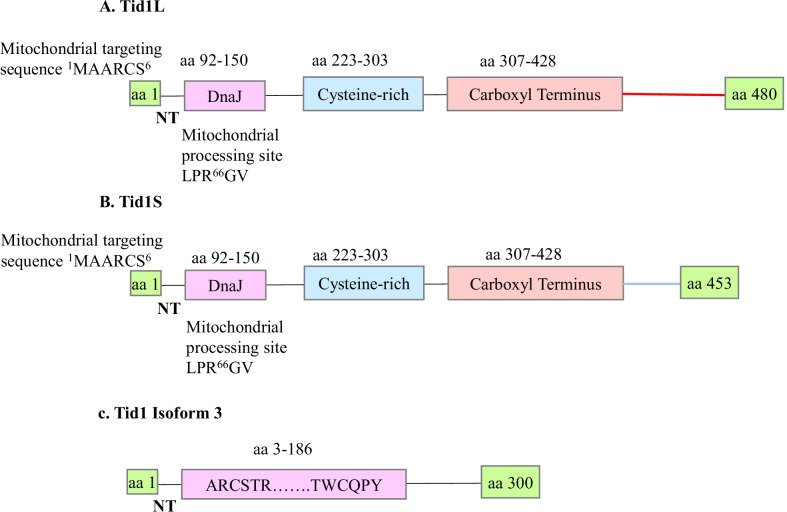
hTid-1 Splice Variants: hTid-1L **A** and hTid-1S **B** comprise distinct functional domains i.e., the amino-terminal (NT) signal sequence (having mitochondrial targeting sequence that targets the protein into the mitochondria), a DnaJ domain, a Cysteine-rich domain resembling a zinc finger repeat sequence (CXXCXGXG), and a non-homologous carboxy-terminal domain (CT). hTid-1L has a set of 33 unique amino acids at its C-terminal end, while hTid-1S has a set of 6 unique amino acids at its C-terminal end. Both hTid-1L and hTid-1S have a predicted mitochondrial processing sequence (LRP-GV) in the NT domain, which is cleaved at amino acid 66 upon entry into the mitochondria. (C). hTid-1 isoform 3 has 300 amino acids and differs from hTid-1L and hTid-1S by a unique sequence of amino acids from 3 to 186

hTid-1 is a 52 kDa protein, and there are three alternately spliced variants of Tid1, the most important of them being Tid1L (43 kDa) and Tid1S (40 kDa) respectively as demonstrated in Fig. 1A and [13]. The two variants differ in their carboxyl-terminus ends, and expression of Tid1L has been found to increase apoptosis while Tid1S has been found to suppress apoptosis in response to both tumor necrosis factor α and DNA-damaging agent mitomycin c [13]. Tid1L contains 33 amino acids that are unique to its C-terminal end (GGSTMDSSAGSKARREAGEDEEGFLSKLKKMFTS) while Tid1S has 6 unique amino acids in its C-Terminal domain (GKRSTGN). Both Tid1L and Tid1S have a predicted mitochondrial targeting sequence in the amino-terminal end and the signature J-domain carrying an HPD Motif important for stimulation of ATPase activity of DnaK-like chaperones [10–12]. The relative ratio of the two proteins has been found to differ in different cell types, for example, while HEK293EBNA (HEK 293 cell line expressing the Epstein-Barr virus nuclear antigen-1) or HEK293T cells have 4–fivefold more hTid-1S than hTid-1L, while the ratio is nearly 1:1 in the Human osteosarcoma, U2OS cells [9].

Figure 1C shows the structure of the third splice variant, commonly called Isoform 3, a 300-base pair long polypeptide with a DnaJ domain and distinct N- and C-terminal domains (NCBI Accession No: NP_001273445.1), however, the presence of the isoform 3 is yet to be experimentally validated.

Pulse-chase experiments have revealed that hTid-1L resides in the cytosol for a much higher duration before mitochondrial transport, while hTid-1S has been found to translocate to the mitochondria much faster [9]. The delayed import of hTid-1L into the mitochondria may be due to its interaction with cytosolic proteins like cytosolic Hsc70, and cytosolic STAT1 and STAT3 proteins. hTid-1L has been found to interact with these proteins via its unique C-terminal domain [9]. It has been found that hTid-1S doesn’t interact with the cytosolic proteins at all [9]. Although a vast majority of hTid-1 localizes to the mitochondria [13, 14], the reported protein interactions and functions of hTid-1 are predominantly non-mitochondrial [15–22]. hTid-1 has been found to interact directly with several cytosolic and nuclear proteins such as E7, an oncoprotein of human papillomavirus [7], UL9, an origin binding protein from herpes simplex virus type 1 [18], the Ras GTPase-activating protein [23], Trk receptor tyrosine kinases [24], Hrfi, a novel protein expressed in esophageal cancers [25] and several other proteins. Recent studies have also shown that hTid-1 helps in the translocation of p53 into the mitochondria under hypoxic conditions, leading to a transcription-independent mitochondrial p53 apoptotic pathway [26].

The two hTid-1 splice variants have been found to play different roles in different kinds of cancers, and are often observed to affect the prognosis in an antagonistic manner. It has been observed that hTid-1 is either reduced or absent in clinical samples from oral cancer patients [27, 28], while in Head and Neck Squamous Cell Carcinomas, hTid-1 has been found to negatively regulate Galectin-7 (which plays an important role in disease progression), by its ubiquitination and subsequent proteasomal degradation [29]. On the contrary, in Non-Small Cell Lung Cancer patients, hTid-1 expression is extremely high in the mitochondrial fraction of the cancerous lesions from Grade IV patients, and that correlated with metastasis of lymph nodes and poor disease prognosis [30]. hTid-1 plays critical roles in a myriad of cellular processes that control, growth, survival, proliferation, and movement.

hTid-1 also plays an important role in the early developmental stages of mammals. Mice deficient in Tid-1 in the heart have been shown to develop dilated cardiomyopathy, progressive respiratory chain deficiency, and decreased copy number of mtDNA [31]. It has also been found that transgenic mice with muscle-specific hTid-1 deletion, display muscular dystrophic syndrome [32]. Owing to its diverse role in critical cellular activities, it is evident that any change in the cellular expression level of hTid-1 causes major imbalances and anomalies, resulting in different diseases and disorders. hTid-1 has also been found to play key roles in cancers, cardiac development, hypertrophies, and neurodegenerative disorders, most notably, Alzheimer’s and Parkinson’s disease [33, 34]. While the focus of the earliest research on hTid-1 was on understanding the interactions of hTid-1 with different proteins in the cell, and its role in cellular signaling pathways, it was only in the past decade that a lot of focus shifted to investigating the role of hTid-1 in the context of different diseases such as cancers, cardiomyopathies, and neurodegenerative diseases. This review aims to reveal the relevance of hTid-1 and its protein–protein interactions in the outcomes of important cellular signaling pathways, and hints at the diseases that result from the varying expression levels of hTid-1 and its interactions with other proteins.

## Main text

### Role of hTid-1 in the cell

**Fig. 2:**
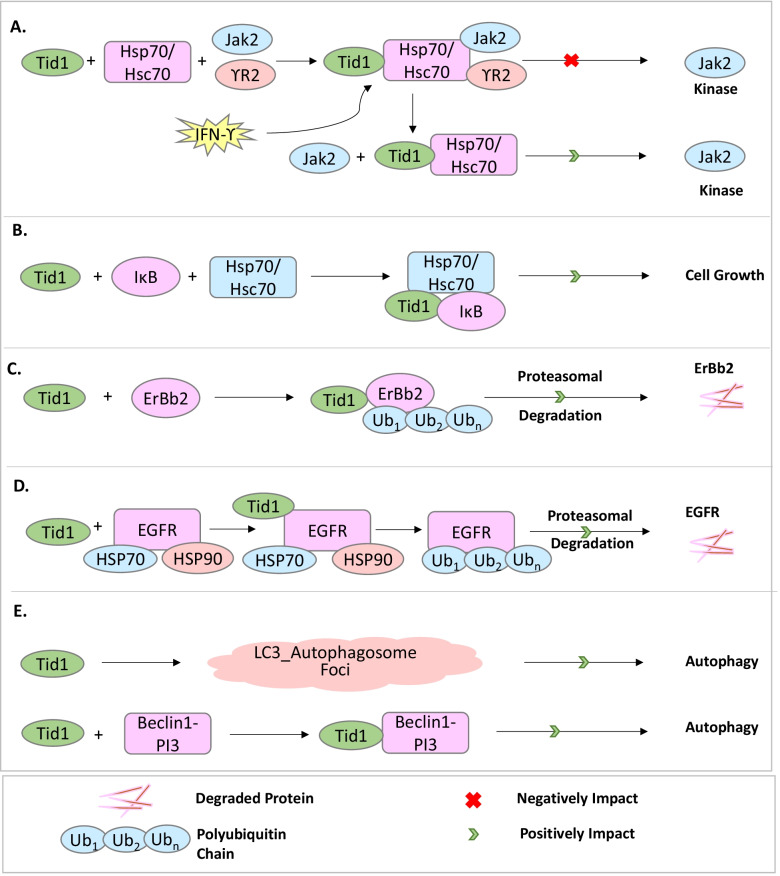
Role of hTid-1 in various cellular processes. **A** hTid-1 acts as a co-chaperone causing conformational changes in Hsp70/Hsc70 which helps it to interact with Jak2 kinases, thereby inhibiting it. **B** hTid-1 along with Hsp70/Hsc70 interacts with IκB and helps in cell growth. **C** hTid-1L causes the attachment of poly-ubiquitin chains to ErBb2 which leads to its subsequent degradation via the proteasomal pathway. **D** hTid-1 causes the attachment of poly-ubiquitin chains to EGFR which leads to its subsequent degradation via the proteasomal pathway. **E** hTid-1 plays an important role in autophagic degradation by interaction with the Beclin1-PI3 kinase class III protein complex

hTid-1 performs a myriad of cellular functions, such as a co-chaperone to Hsp70, as an E3-ligase to several interacting proteins eventually leading to their degradation via the 26S proteasome, as an important protein agent leading to autophagic degradation of cellular proteins, in several signaling pathways of the cell (such as the Wnt, Trk, and Agrin signaling pathways), and the early developmental stages of mammals, as has been explained extensively in Fig. 2. Co-chaperones act as catalysts leading to the hydrolysis of ATP to ADP on their respective chaperones which allows them to undergo important conformational changes, thereby allowing them to either bind to their substrates with high affinity or aid in the release of the misfolded proteins post their proper folding [35]. hTid-1 is a co-chaperone for Hsp70 as shown in Figs. 2A, B, and is widely expressed in a variety of organisms from bacteria to humans [36, 37]. hTid-1 interacts with the Hsp70 proteins in the cytosol (Hsp70/Hsc70) or the mitochondria (mtHsp70) through its conserved DnaJ domain, thereby modulating the activities and substrate binding specificities of the Hsp70 proteins [13, 38].

Another important function of hTid-1 is its role in the degradation of misfolded and aberrant proteins as explained in Figs. 2C, D, both via the 26S proteasome (as an E3 Ligase) [39, 40] or via the autophagic pathway of the lysosomes [41]. Degradation via the 26S proteasome requires the substrate protein to be ubiquitinated, which is essentially the covalent attachment of a series of small 76-amino acid proteins called ubiquitin as a post-translational modification to the proteins that need to be degraded [42]. Attachment of polyubiquitin chains tag the substrate proteins for eventual degradation [43] and the entire process requires an ATP-dependent enzymatic cascade initiated by a ubiquitin-activating enzyme E1, a ubiquitin-conjugating enzyme E2 [44, 45], and a ubiquitin Ligase enzyme E3 [42], hTid-1 functions as an E3 ligase for certain proteins in the cell such as ErbB2, EGFR and Galectin-7 [29, 30, 46]. These interactions of hTid-1 help in controlling major aspects of cancer cell growth.

Also, hTid-1 is an important mediator of the degradation of proteins via the autophagic pathway in the lysosomes as demonstrated in Fig. 2E [41]. Autophagy is the internal self-digesting mechanism of the cell of degradation and removal of damaged cellular organelles and long-lived and misfolded proteins via the lysosomes [47, 48]. Classic to the process of autophagy is the evolutionarily conserved Atg (autophagy-related) proteins, which were predominantly identified in yeast [49–51]. The initiation of the process of autophagy essentially requires two complexes, (1) A complex which requires the Class III PI3K proteins, Vps34, Atg6/Beclin1, Atg14, and Vps15/p150.73. The other complex includes a serine/threonine kinase Atg1 which requires the function of the autophagy proteins, Atg13 or Atg8 and Atg 17. In mammals that do not have Atg13, Atg1 associates with the Atg8 orthologues, LC3 (microtubule-associated protein light chain 3), GATE-16 (Golgi-associated ATPase enhancer of 16 KDa), and GABARAP. The kinase activity of Atg1 requires the function of two other autophagy proteins, that is, Atg13 or Atg8 and Atg17. In mammals, which do not contain Atg13, Atg1 was found to associate with the Atg8 orthologues, LC3 (microtubule-associated protein light chain 3), GATE-16 (Golgi-associated ATPase enhancer of 16 KDa), and GABARAP (G-amino butyric acid type A receptor-associated protein). Thereafter, the elongation phase requires two conjugation pathways which are the Atg8/MAP/LC3/GABARAP/GATE-16 and Atg12 systems. As the autophagosome formation is completed, the Atg proteins disassociate from it and the autophagosome fuses with the lysosome or the endosome [49–51]. While the role of some of the stress-induced chaperones like Hsp70 in autophagosome-forming macroautophagy has been studied under different stress conditions, not much was known about the role of their co-chaperones in these processes. hTid-1, a DnaJ co-chaperone has been shown to be a key mediator of the macroautophagy pathway by the formation of the LC3 + autophagosome foci [41].

Similarly, hTid-1 is also a critical component of several signaling pathways of the cell like the Wnt signaling pathway, a conserved pathway in metazoan animals [52], which stimulates several intracellular signal transduction cascades including the canonical or Wnt/β-catenin dependent pathway and the non-canonical or β-catenin-independent pathway [53]. Further, hTid-1 is important to the functioning of the Trk signaling pathway which affects neuronal survival and differentiation through different signaling cascades [54, 55]. Since hTid-1 plays such important and diverse roles in the cell, it would be interesting to find out its interacting protein partners and how these interactions regulate cell fate. Some of the studies are as follows:

#### As a co-chaperone

hTid-1 is an important co-chaperone to Hsp70 which regulates important aspects of the cellular machinery like protein folding, chaperoning functions, and protecting cells amid several physiological stresses [56, 57]. hTid-1 binds to Hsp70 via its conserved DnaJ domain and actively helps to regulate the specificity and activity of their substrate proteins [8, 9]. The interaction between hTid-1 and Hsp70 was shown to be important in inhibiting the kinase activity of Jak2 kinases. The Jak family of protein tyrosine kinases control the signaling of several polypeptide ligands which include growth factors, erythropoietin, several cytokines, and interferons [58, 59]. hTid-1 has been found to interact with the non-receptor tyrosine kinase protein, Janus Kinase 2 (Jak2 kinases) [15, 60]. Activation of Jak2 is implicated in causing leukemia, and hence its activation needs to be highly regulated. hTid-1 acts as an important negative regulator of the JAK-STAT pathway [15]. The Jak2 kinases are associated with downstream proteins including signal transducers and activators of transcription (STATs) which translocate to the nucleus upon being phosphorylated and regulate organismal development and homeostasis [60]. hTid-1 interacts with Jak2 and also with the human interferon-ϒ (HuIFN-ϒ) receptor subunit IFN-ϒR2. Jak2 and ϒR2 associate with hTid-1 and Hsp70/Hsc70 to form a complex, hTid-1 functions like a co-chaperone causing a conformational change in Hsp70/Hsc70 which helps it to interact with Jak2 causing inhibition of the kinase activity of Jak2. In the presence of IFN-ϒ, Hsp70/Hsc70 and later hTid-1 are released from the complex leading to the activation of the kinase function of Jak2 [15].

#### In proteasomal degradation

Poly-ubiquitination of proteins mediated by E3-ligases generates signals for the degradation of such proteins via the 26S proteasome. hTid-1 has been found to act as an E3-ligase for several interacting proteins such as ErbB2 and EGFR that play important roles in Breast and Lung cancers respectively [30, 39, 46, 61]. hTid-1 causes the attachment of poly-ubiquitin chains to its client proteins which leads to their degradation via the proteasomal pathway. The transmembrane glycoprotein, ErbB2/HER2 receptor overexpression has been found to be an important biomarker for a range of different solid human tumors such as mammary and ovarian tumors and has been associated with a poor prognosis. Its cytoplasmic domain which has docking sites for several signaling molecules sends out mitogenic signals to cells controlling activities such as activation of numerous signaling pathways. These pathways affect important cellular responses affecting proliferation, differentiation, survival, and apoptosis. Intracellular hTid-1 interacts with the ErbB2 signaling domain, causing its proteasomal degradation via polyubiquitination, thereby helping in the attenuation of mitogenic signaling from ErbB2 in cancer cells [30].

Another protein that interacts with hTid-1 through the DnaJ domain is the Receptor Tyrosine Kinase (RTK), and EGF Receptor (EGFR) which is a key driver protein of lung adenocarcinoma by regulating tumorigenic processes including invasion, angiogenesis, and apoptosis. The HSP90 chaperone along with co-chaperones HSP70 and HSP40 helps in protein-folding, activation, or degradation of EGFR. hTid-1L interacts with the EGFR/HSP70/HSP90 and causes its ubiquitination and subsequent proteasomal degradation, thereby downregulating EGFR signaling [61]. hTid-1 also interacts with the Nuclear Factor kappa-light-chain-enhancer of activated B bells, commonly called, NF-κB, which actively regulates the expression of many genes involved in processes like oncogenesis, inflammation, immunity, and anti-apoptotic response [62, 63]. In resting cells, NF-κB is held back inside the cytoplasm by its inhibitor IKB proteins such as IκBα and IκBβ through the formation of an inactive NF-ΚB-IκB protein complex. Once activated, IκB is phosphorylated, polyubiquitinated, and proteasomally degraded, which is when NF-κB is released from the protein complex and is imported into the nucleus, causing the activation of expression of different genes. hTid-1 represses the activity of NFκB through interactions with the IKK complex and IκB, hence modulating cell growth and death [64].

#### In autophagic degradation

hTid-1 plays an important role not only as an E3-Ligase for different interacting proteins leading to proteasomal degradation but is also a critical part of canonical macroautophagy pathways. Studies by Niu et al. [41] have shown that ectopic expression of hTid-1 facilitates the formation of the LC3_autophagosome foci containing Beclin1-PI3KC3 (PI3 kinase Class III) during the vesicular nucleation stage. The two domains of hTid-1 that are essential for the autophagy process are the N-terminal domain which mediates the binding of hTid-1 to Beclin-1 and the J-domain which majorly executes the induction of autophagy. It was already known that stress-induced Hsp70 interacts with Beclin-1, which is a key component of the autophagy complex along with PI3KC3 [40]. However, Niu et al. [41] also showed that hTid-1 initiates autophagy independent of its association with Hsp70. Domain deletion mutants lacking a J-domain which is critical for the interaction of hTid-1 with Hsp70 could also initiate autophagy by retaining their ability to enhance the LC3-II levels. Also, hTid-1 and Hsp70 were found to initiate apoptosis under different stresses, hTid-1 under nutrient deprivation or Rapamycin-induced canonical macroautophagy, whereas Hsp70 under hyperthermia-, or heavy metal-induced autophagy. Autophagy is an important process in the cell for the maintenance of chromosomal stability and plays an important role in tumor suppression [65]. In the context of that, it would be interesting to conduct more studies on the autophagy-inducing function of hTid-1 in correlation to its role in oncogenesis, which would help to develop cancer therapies in the future with hTid-1 being an important protein of interest.

#### In cellular signaling pathways

**Fig. 3:**
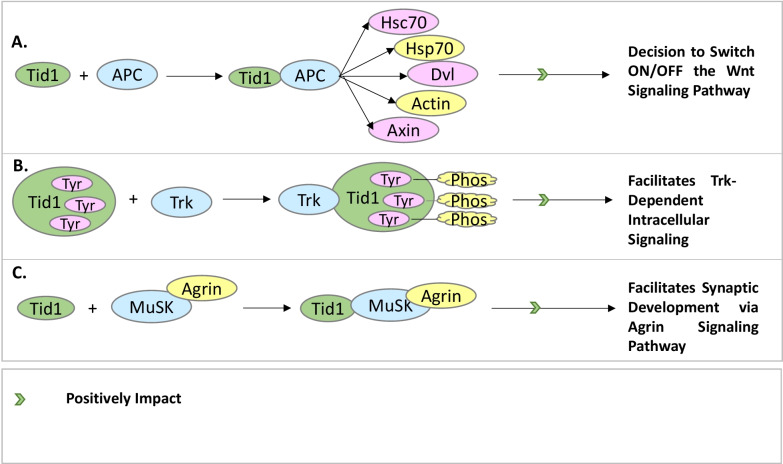
Overview of hTid-1 interactions in cellular signaling pathways. **A** hTid-1 interacts with the APC protein complex which further interacts with other proteins to regulate the Wnt signaling pathway. **B** hTid-1 interacts with Trk kinase which causes the phosphorylation of the tyrosine residues of hTid-1, facilitating Trk-dependent intracellular signaling. **C** hTid-1 interacts with the MuSK and Agrin proteins, which facilitates synaptic development via the Agrin signaling pathway

It has been reported that hTid-1 has been found to play a critical role in the Wnt signaling pathway as explained in Fig. 3A. The Wnt family comprises of nineteen secreted glycoproteins which are critical to the evolutionarily conserved Wnt signaling pathway that regulates cell fate determination, cell migration, cell polarity, neural patterning, and organogenesis during embryonic development [52]. The main categories of the Wnt signaling pathway are the canonical Wnt pathway, the non-canonical planar cell polarity pathway, and the non-canonical Wnt/calcium pathway. Key to the functioning of the canonical Wnt pathway is the accumulation of beta-catenin (β-catenin) [66] which translocates into the nucleus and acts as a transcriptional coactivator of transcription factors belonging to the TCF/LEF family. The degradation of beta-catenin in the Wnt canonical pathway is caused by a destruction complex harboring Adenomatous Polyposis Coli (APC) that targets it for ubiquitination and subsequent proteasomal degradation [66, 67]. The Tid50/Tid48 cytosolic splice variants of hTid-1 and the Adenomatous Polyposis Coli (APC) tumor suppressor associate with each other in different tissue samples such as colorectal cancer cells, normal colon epithelium, and mouse NIH3T3 fibroblasts, and it has been found that the Armadillo domain (ARM) containing N-terminal region of APC is sufficient for interaction with hTid-1 molecules [68]. While the formation of the hTid-1-APC complex is not directly associated with the degradation of beta-catenin by APC, however, it has been found that hTid-1 and APC form complexes together with other partners which include Hsc70, Hsp70, Dvl, Actin, and Axin and help in maintaining the availability of APC for its specific roles in the Wnt signaling pathway [68]. This is critical for cells to confirm the decision of whether to switch the cascade ON/OFF and thereby regulate the initiation of the proliferation of cells [68].

hTid-1 has been found to associate with the Trk receptor tyrosine kinases (Fig. 3B) which regulate synaptic strength and plasticity in the mammalian nervous system [54, 55]. hTid-1 interaction with the Trk receptor tyrosine kinases regulates the nerve growth factor (NGF)-induced neurite growth in PC12-derived nnr5 cells. It was shown that the carboxyl-terminus of hTid-1 binds to Trk at the activation loop, and that hTid-1 is phosphorylated at tyrosine residues by Trk in both transfected cells and yeast. These studies demonstrated that the interaction between hTid-1 and the Trk receptor tyrosine kinases facilitates Trk-dependent intracellular signaling [24].

hTid-1 has also been found to bind to the cytoplasmic domain of muscle-specific kinase (MuSK) as highlighted in Fig. 3C. MuSK is a key component of the agrin receptor and acts as an indispensable molecule of the agrin signaling pathway which is important for synaptic development. Agrin acts via a membrane receptor complex harboring muscle-specific kinase MuSK which is a receptor Tyrosine Kinase (RTK) [69, 70]. For synaptic transmission, clustering of acetylcholine receptors (AChRs) at the post-synaptic membrane is critical and agrin facilitates the process for efficient synaptic transmission [71, 72]. Specifically, hTid-1S has been found to bind to the juxtamembrane region of MuSK in two-hybrid experiments [73]. hTid-1 has been found to be co-localized with agrin-induced and spontaneous AChRs in cultured myotubes, and in muscles, hTid-1 co-localizes with AChR clusters throughout the morphological changes that occur in post-synaptic membranes during maturation, denervation, and reinnervation [73]. Thus, hTid-1 plays an important role in the induction and maintenance of high-density AChR clusters and mediates agrin signaling at the Neuromuscular Junction (NMJ).

### Mitochondrial translocation and homeostasis

Mitochondria carry out several important functions of the eukaryotic cell, however, only a very small proportion of the mitochondrial proteins are synthesized by the mitochondrial ribosomes [74]. The other proteins responsible for different mitochondrial functions are synthesized in the cytosol and imported inside the mitochondria by the translocase proteins in the inner membrane of the mitochondria (TIM complexes), or the translocase proteins in the outer membrane of the mitochondria (TOM complexes). These proteins play important roles in cross-talk with the cytosol, uptake of metabolites, lipids, or metal ions, and with the regulation and execution of apoptosis [75–78]. Mitochondrial hTid-1 helps in maintaining the integrity of mitochondrial DNA (mtDNA) and a homogeneous distribution of membrane potential [9, 13, 80]. It is reported that the hTid-1-Mortalin complex acts as the Hsp40-Hsp70 chaperone system in the mitochondria and helps in the normal distribution of electrochemical potential (Δψ) across the mitochondrial membrane, where the DnaJ domain of hTid-1 plays a critical role. hTid-1S has been found to rapidly translocate into the mitochondria [9], while reconstitution of hTid-1S, but not hTid-1L in hTid-1-depleted cells has been observed to restore Δψ, thereby signifying the fact that hTid-1S and hTid-1L play distinctly functional roles in the mitochondria. Upon hTid-1-silencing, the mitochondrial Complex I protein NDUFS3 shows a punctuated submitochondrial distribution and selectively colocalizes with the hyperpolarized regions of the network. The two subunits of Complex-I, i.e., NDUFS3 and NDUFA9 of the Electron Transport Chain (ETC) are found in the detergent-insoluble fraction, which hints at the prospect of aggregated Complex-I being responsible for hyperpolarization of the mitochondrial membrane [80]. Mitochondrial membrane potential is the result of electron transport-coupled proton extrusion into the intermembrane space by complexes I, III, and IV. Thus, upon hTid-1 silencing, aggregation of Complex-I in the sub-mitochondrial foci results in the creation of hot spots in these regions. Over-expression of hTid-1 has been found to resolubilize the Complex-1 aggregates indicating that hTid-1 plays a major role in the maintenance of mitochondrial membrane potential homogeneity and functions as a co-chaperone which helps to prevent Complex-I aggregation and elicits a stress response to ATP synthase inhibition [80]. Cheng et al. [32] reported that hTid-1 actively participates in skeletal muscle myogenesis by impairing the mitochondrial activity of muscle cells, due to which muscle cell apoptosis occurs. However, the complete mechanism of reduction in ATP levels is still not explored.

hTid-1 plays a crucial role in the mitochondrial translocation of various other proteins, including proteins that are involved in different cancers. One important example is p53, which induces apoptosis owing to its tumor-suppressive role. It was recently reported that a transcription-independent mitochondrial pathway of apoptosis also exists [81]. However, not much was known about the translocation of p53 into the mitochondria. hTid-1 complex formation with p53 under hypoxic conditions was recently studied and it was observed that the complex translocates into the mitochondria where it induces the mitochondrial pathway of apoptosis. A critical observation states that the translocation of p53 into the mitochondria requires both the N-terminal mitochondrial sequence and DnaJ domain of hTid-1 and subsequently causes apoptosis via the intrinsic pathway. It was also found that when hTid-1 was overexpressed in mutant p53-expressing cancer cells such as T47D (p53 ^mt−L194F^), SK-BR-3 (p53 ^mt−R175H^), BT474 (p53 ^mt−E285K^) and the glioma cell line, U373 (p53 ^mt−R273H^), which lacked transcriptional activity, it restored the localization into mitochondria and the pro-apoptotic activities of mutant p53 [26]. These results suggest the chaperoning of mutant p53, though it would be interesting to find out the pathway resulting in the same. Further, far-western analyses by Trinh et al. [82] led to the conclusion that hTid-1 directly interacts with p53 to aid in its mitochondrial localization and its DnaJ domain is necessary for the interaction, while either of its N- or C-terminal domains is sufficient for the interaction. Their study also showed that silencing of hTid-1 by short hairpin RNA (shRNA) in breast cancer cells led to the complete barring of p53 entry into the mitochondria, resulting in impediment of apoptosis under low oxygen and genotoxic stresses.

Similarly, another important protein to be studied in the respect of hTid-1 is the Epidermal Growth Factor Receptor (EGFR) which is the major driver of Non-Small Cell Lung Cancer (NSCLC) [83]. It was shown that the accumulation of EGFR in the mitochondria increases the metastasis of NSCLC cells [84]. Wang et al. [86]. reported that the DnaJ domain of hTid-1S is essential for the transport of EGFR into the mitochondria through the mtHSP70 transportation pathway. Increased levels of hTid-1S and EGFR were obtained in the mitochondrial fractions of cancerous lesions of Grade IV NSCLC patients, which can be correlated with the poor overall survival of these patients [85]. Later Wang et al. [86] reported hTid-1 as an important prognostic marker in the gastric cancer cells which showed decreased cell proliferation, colony, and tumor sphere formation and chemoresistance. However, hTid-1 knockdown consequently decreases the mitochondrial DNA (mtDNA) copy number but did not consistently affect the mitochondrial content, respiratory function, and ROS production.

Thus, it can be observed that hTid-1 plays a very critical role in the maintenance of homeostasis in the mitochondria via the regulation of its two important aspects, that is, the mitochondrial DNA content and mitochondrial membrane potential. It is also an important translocase protein for several interacting proteins that play important roles in different cancers, and the expression levels of hTid-1 play an important role in the prognosis of such cancers.

### Role of hTid-1 in cancer cells

#### Regulating cancer-associated proteins

**Fig. 4:**
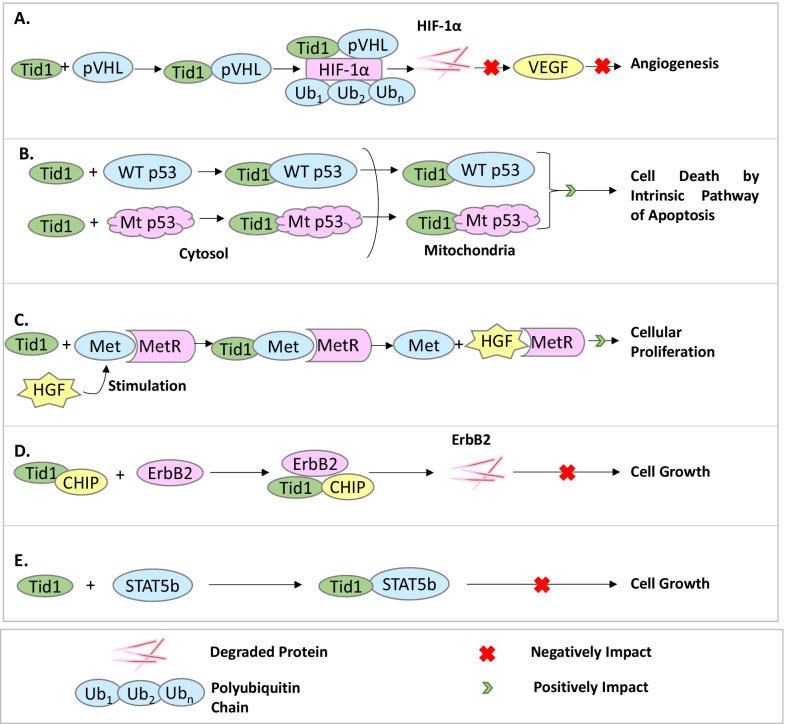
hTid-1 in the regulation of cancer-associated proteins. **A** hTid-1enhances the attachment of pVHL with HIF-1α which increases the attachment of poly-ubiquitin chains to HIF-1α, and its subsequent degradation via the proteasomal pathway. **B** hTid-1 interacts with both Wild-Type and mutant p53, causing the translocation of p53 into the mitochondria and subsequent apoptosis via the intrinsic pathway. **C** Upon HGF stimulation, hTid-1 interacts with the Met protein causing its dissociation from the Met Receptor, and an increase in cellular proliferation. **D** hTid-1 along with CHIP causes the degradation of ErBb2. **E** hTid-1 interacts with STAT5b and stops cell growth

Hypoxia is the state in which insufficient oxygen is available in the cell or at the tissue level to maintain adequate homeostasis. Hypoxia has been found to be an important factor favoring the growth of cancer cells. HIF-1α is an important protein involved in the hypoxic responses exhibited by cancer cells. The tumor suppressor, Von Hippel-Lindau protein (pVHL) interacts with HIF-1α, causing its ubiquitination and proteasomal degradation [87], thereby causing the inhibition of angiogenesis. Figure 4A shows how hTid-1L interacts directly with pVHL and enhances the interaction between pVHL and HIF-1α, leading to the proteasomal degradation of HIF-1α, hence reducing the expression levels of Vascular Endothelial Growth Factor (VEGF) and subsequently inhibiting angiogenesis of tumors [88].

Another important factor at the focal point of any cancer is the unlimited proliferation of the cells, and very less or no apoptosis because of the mutant p53 in these cells which loses its transcriptional pro-apoptotic activity and hence doesn’t initiate apoptotic pathways. Many tumors have been found to hold p53 mutations, however, it remains controversial as to whether tumor cells with mutant p53 have an impaired p53-mediated apoptosis pathway [26]. P53 mutants such as R273H and R175H have been found to lose their anti-apoptotic activity [89, 90]. Ahn et al. [26], showed that the interaction of hTid-1 with p53 causes the mitochondrial translocation of the complex, which results in apoptosis of cancer cells. In cancer cells with mutant p53, apoptosis is severely impacted. The over-expression of hTid-1 has been seen to cause translocation of the p53-hTid-1 complex to mitochondria and eventual apoptosis of these cells through the intrinsic pathway rather than through the nuclear processes as has been explained in Fig. 4B.

Another studied cancer cell signaling system is MetR, where hTid-1 plays an important role as highlighted in Fig. 4C. The c-Met receptor Tyrosine Kinase (MetR) is found to be periodically overexpressed, and constitutively phosphorylated in a variety of human malignancies. It has been observed that upon Hepatocyte Growth Factor (HGF) stimulation, hTid-1 binds to unphosphorylated MetR and dissociates it from its receptor. HGF acts as a ligand of the Met Receptor, and upon activation via HGF, the Met Receptor causes amplification of cell motility, proliferation, survival, and adherens junction disruption. Over-expression of hTid-1S has been found to enhance the activity of MetR Kinase, causing a subsequent amplification in HGF-mediated cellular migration, however, it had not been observed to directly affect cell proliferation. These findings suggest that hTid-1S plays a critical role in the modulation of MetR signaling, and hTid-1S binding to MetR stabilizes the receptor, and as a result influences the conformational changes taking place during the catalytic cycle, thereby promoting the activation of kinases [91]. Similarly, an important protein in Breast Cancer signaling, ErbB2 has also been found to be regulated by hTid-1 as pointed out in Fig. 4D. Carboxyl terminus of heat shock cognate 70 interacting protein (CHIP), has also been found to efficiently downregulate ErbB2 in vitro, quite similar to h-hTid-1. ErbB2, CHIP, and hTid-1 have also been shown to interact with each other. When the expression and correlation between CHIP, hTid-1 and ErbB2 were analyzed using immunohistochemistry (IHC) and immunoblotting assays in 183 breast cancer histology sections, which included 30 fresh tissue specimens, it was found that hTid-1 and CHIP positively correlate with each other and inversely with ErbB2 and that hTid-1 and CHIP act in synergy to degrade ErbB2 in vitro [92]. Further, in hematopoietic cell lines, the DnaJ domain of hTid-1 has been seen to be involved in interactions with STAT5b (Fig. 4E), negatively regulating its expression and transcriptional activity causing suppression of the growth of cells that have been transformed by the oncogenic form of STAT5b, but not STAT5a [93]. Thus, it can be stated that hTid-1 is an important component of cancer cell signaling and controls several different aspects of cancer cell growth, proliferation, survival, and apoptosis.

#### Regulating cancers

hTid-1 has been found to be an important tumor suppressor in several cancers such as Head and Neck carcinoma, oral and lung cancers, and in tumors of glial origin among several others. The expression levels are found to be perpetually low in these cancers. The tumor suppressor roles of hTid-1 are also reported in osteosarcoma cells where hTid-1 silencing offers an advantage against apoptosis [13]. By depletion of Interleukin-8 (IL8), hTid-1 has been found to be critically important in curbing the migratory potential of cancer cells, which suggests that hTid-1 may also play an important role against cancer metastasis [21]. Further, hTid-1 has been found to interact with the von Hippel-Lindau protein to destabilize Hypoxia Inducible Factor 1-alpha (HIF-1α) in sarcoma and cervical cancer cells, thereby regulating angiogenesis [65]. As shown in Fig. 4, hTid-1 plays a significant role in regulating the key-biomarker proteins of different cancers which suggests its importance as a therapeutic target. Researchers have explored the expression level of hTid-1 in cancers and stated that the expression levels of hTid-1 are found to be low in most cancers as demonstrated in Table 1, however, it is the opposite in some cases as explained further.

**Table 1:** The expression levels of hTid-1 in different patient cancer samples and how it affects the prognosis of those cancer patients

S. No	Disease	Patient samples	Expression	Processes affected	Survival rate	References
1	Head and neck squamous cell carcinoma	47 HNSCC biopsies	Low	Tumor differentiation	Poor	Chen et al. [27]
2	Lung adenocarcinoma	55 patient tumor samples	High	Tumor growth	Better	Chen et al. [61]
3	Non-small cell lung cancer	20 surgically resected Tumor samples	Low	–	Poor	Chen et al. [61]
4	Non-small cell lung cancer	NSCLC patient samples	Low Tid1L	Tumorigenesis	Poor	Chen et al. [39]
5	Non-small cell lung cancer	Tumor samples from Stage-IV NSCLC patients	High Tid1S	Metastasis and invasion	Poor	Wang et al. [30]
6	Hepatocellular carcinoma	210 surgically resected HCC specimens	Low	Recurrence of HCC	Poor	Chen et al. [106]

**Fig. 5:**
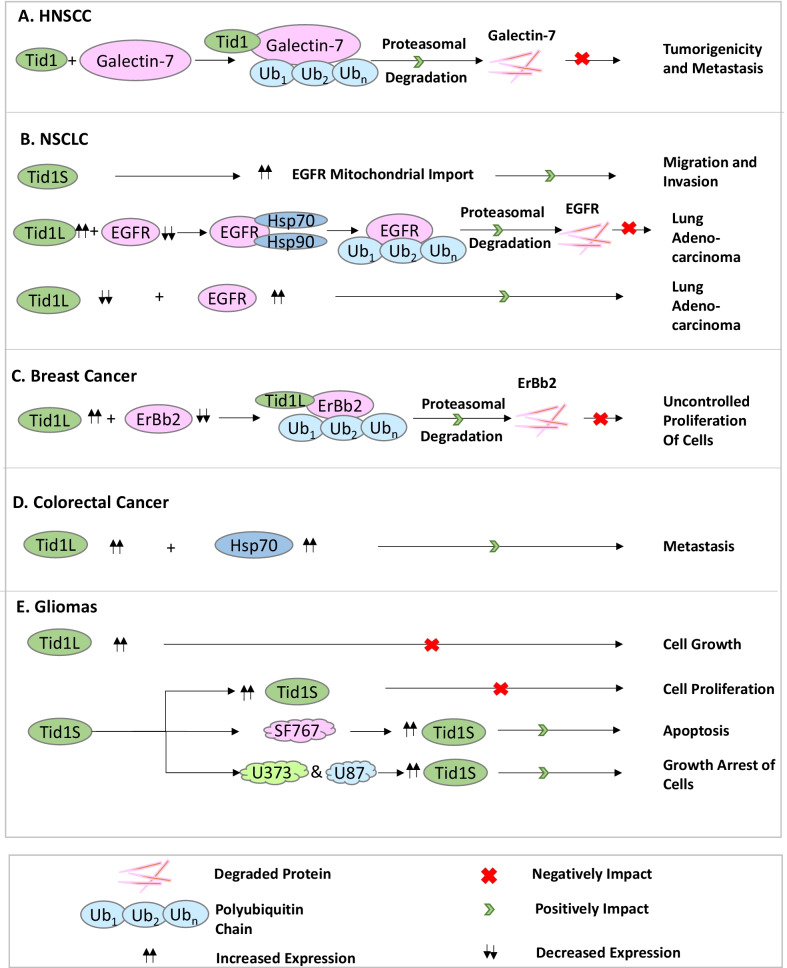
hTid-1 in the regulation of cancers. **A** In Head and Neck Squamous Cell Carcinoma cells, hTid-1 Interacts with Galectin-7, causing the attachment of poly-ubiquitin chains to Galectin-7 and its subsequent degradation via the proteasomal pathway. **B** In Non-Small Cell Lung Cancer cells, hTid-1S increases the mitochondrial import of EGFR which increases the migration and invasiveness of these cells, while hTid-1L causes the attachment of poly-ubiquitin chains to EGFR and its subsequent degradation via the proteasomal pathway. **C** In breast cancer cells, hTid-1L causes the attachment of poly-ubiquitin chains to ErBb2 and its subsequent degradation via the proteasomal pathway. **D** In Colorectal cancer cells, increased expression of hTid-1L and Hsp70 enhances the metastasis of these cells. **E** In Gliomas, increased expression of hTid-1L stops cell growth, while increased expression of hTid-1S has been observed to inhibit cell proliferation, causing apoptosis of SF767 cells and growth arrest of U373 and U87 cells

Head and Neck Squamous Cell Carcinomas (HNSCC) are the sixth-most prevalent cancers worldwide and have been found to be linked to environmental carcinogens [27]. hTid-1 has been found to play the role of a tumor suppressor in these cases as shown in Fig. 5A. Chen et al. [27] analyzed the protein levels of hTid-1 in the biopsies of 47 HNSCC and NCMT (non-cancerous matched tissues) pairs by Immunohistochemistry studies, and the hTid-1 expression was found to be inversely correlated with tumor differentiation. Significantly reduced to almost no hTid-1 was detected in the poorly differentiated carcinomas. Assessment of the rate of survival after cancer treatment is a major step in prognosis. Kaplan–Meier survival curves are the simplest and best way to the compute the fraction of survival of subjects over time after a certain kind of treatment These curves take into account the difficulties associated with subjects or situations and involve a probability-mediated approach to take into account these factors that are referred to as censored observations [94]. Kaplan–Meier survival analysis curves in HNSCC pointed out that patients with higher expression of hTid-1 had better overall survival rates than the ones with lower or undetectable expression of hTid-1. They also showed that ectopic expression of hTid-1 in oral cancer cell lines with reduced expression of hTid-1 inhibited the capability of cell proliferation, migration, invasion, and anchorage-dependent growth in these cells. The fact that depletion of hTid-1 in these cells by RNA interference enhanced cell proliferation, cell migration, and cell invasion, and also protected the cells from stress-induced apoptosis, goes on to prove that hTid-1 harbors a tumor-suppressive function in oral cancer cells in vitro*.* The outcomes of research done by Demers et al. in [28, 95, 96] reported that the expression of Galectin-7 which belongs to the β-galactoside-binding lectin family is enhanced in aggressive cancers and results in increased metastasis and reduced survival. The analysis of hTid-1 expression in 56 HNSCC tissue sections showed weak staining of Galectin-7 in tissues stained strongly for hTid-1. This proves that hTid-1 and Galectin-7 expressions are inversely correlated with each other. Kaplan–Meier survival curves have demonstrated higher lymph node metastasis and reduced survival for patients with lower expression of hTid-1 and higher expression of Galectin-7. The HNSCC tissues that stained weakly for hTid-1, exhibited strong staining for nuclear Galectin-7, while tissues that stained strongly for hTid-1 had a higher proportion of cytoplasmic Galectin-7. In patients with a stronger nuclear Galectin-7 staining, as compared to cytoplasmic Galectin-7 staining, survival was found to be poor. Studies show that hTid-1L negatively regulates Galectin-7 via N-linked glycosylation which promotes degradation of Galectin-7 by poly-ubiquitination which neutralizes tumorigenicity and metastasis of HNSCC [29]. The poor prognosis of HNSCC is attributed to the high levels of metastasis of these cancers [97], and so understanding the molecular basis via which metastasis happens in HNSCC can help in developing better treatment strategies for these cancers.

Non-Small cell lung cancers have been found to be the dominant form of lung cancers which constitute a leading cause of cancer deaths worldwide [98] and the role of hTid-1 in these cancers is explained in Fig. 5B. Chen et al. [61] performed mRNA analyses by quantitative RT-PCR in 20 surgically resected paired samples of tumor and adjacent normal tissues of patients with NSCLC. The expression levels of both hTid-1L and hTid-1S were found to be lower in the tumors than in the adjacent tissues. The EGF receptor (EGFR), a Receptor Tyrosine Kinase (RTK), a key driver molecule of lung adenocarcinomas [99], and its expression has been found to negatively correlate with hTid-1 expression levels in both lung adenocarcinoma cell lines, and in paired tumor and adjacent normal tissues from 55 patients with adenocarcinoma. Survival analysis studies by this group have shown that higher expression of hTid-1L and lower expression of EGFR are associated with increased survival possibility of patients with lung adenocarcinoma. hTid-1 interacts with EGFR/HSP70/HSP90 via its DnaJ domain and induces poly-ubiquitination resulting in subsequent proteasomal degradation of EGFR, thereby downregulating its expression levels and hence acting as a deterrent in the progression of lung adenocarcinomas [61]. While hTid-1L acts as a tumor suppressor in lung adenocarcinomas, it was of interest to find out the molecular mechanisms that regulated the alternate splicing of hTid-1. Therefore, Chen et al. [39] later reported that heterogeneous nuclear ribonucleoproteins (hnRNP) A1 and A2 are the two splicing factors participating in alternate splicing of hTid-1 and are over-expressed in lung cancers. RNAi-mediated depletion of both hnRNP A1/A2 (but not single depletion of either) was found to amplify hTid-1L expression which causes the inhibition of cell proliferation and attenuation of EGFR signaling. These findings suggested that alternative splicing of exon 1 in the hTid-1 pre-mRNA is facilitated by hnRNP A1 or A2, leading to suppression of hTid-1L expression and allowing EGFR-related signaling to facilitate NSCLC tumorigenesis. Increased expression of hnRNP A1 and A2 and EGFR decreases the expression level of hTid-1L in NSCLC patients and presents a poorer prognosis [39]. Stage IV NSCLC patients possess high levels of hTid-1S and EGFR in the mitochondrial sections of the cancerous lesions, causing lymph node metastasis and poor life expectancy. It is demonstrated that hTid-1S facilitates the higher mitochondrial import of EGFR and promotes metastasis-related activities, i.e., migration and invasion of NSCLC cells. This suggests that while hTid-1L majorly has a tumor-suppressive role, hTid-1S acts as a key driver of metastasis, hence acting as an oncogene [30].

As shown in Fig. 5C, in breast cancer studies, hTid-1 has been found to regulate the Erb-B2 Receptor Tyrosine Kinase 2, commonly called the ErbB2/HER2 receptor, by acting as an E3 ligase for the protein, thereby facilitating attachment of poly-ubiquitin chains, leading to subsequent proteasomal degradation of the receptor [46]. The ErbB2/HER2 receptor, which is frequently over-expressed in mammary and ovarian tumors, presents an unfavorable prognosis for these cancers. hTid-1 has been found to be over-expressed in human mammary carcinomas, causing suppression of ErbB2 expression, leading to inhibition of ErbB2-mediated tumor progression. It is observed that in ErbB2-overexpressing carcinoma cells, the co-chaperone activity and regulation of Hsp70 by hTid-1 plays an important role in preventing the uncontrolled proliferation of cells by actively decreasing the expression of ErbB2, thereby resulting in the suppression of the ErbB2-dependent cancerous signaling and tumor progression [46].

Similarly, hTid-1 expression levels are found to play a key role in the progression of colorectal cancers as explained in brief in Fig. 5D. Sporadic and inherited colon tumors both arise from precancerous polyps or adenomas sequentially [100], via a chain of genetic alterations in the evolutionarily conserved tumor suppressor genes and oncogenes, each associated with well-defined morphological changes [101]. The adenomatous polyposis coli (APC) protein is critical to the functioning of the Wg/Wnt signaling cascade, which is activated in the presence of the Wg/Wnt signaling molecule and helps to regulate the expression of β-Catenin (core protein of the cadherin protein complex whose stabilization is essential for the activation of the Wg/Wnt pathway). The TCF/LEF transcription factors engaged in complex formation with β-Catenin initiate the expression of target genes such as cyclin D1 or c-myc, which regulate cell proliferation [102–104]. An increase in Hsp70 which is a direct ligand of hTid-150/hTid48, along with an increase in hTid-1 was found to actively enhance the metastatic potential of colorectal tumors [105]. Kurzik-Dumke et al. [105] examined the expression of hTid-1 in untreated primary human tumors and observed a high expression of hTid-1 and alterations in their localization, i.e., the compartmentalization of both molecules in the normal colon epithelium was found to be absent accompanying the loss of differentiation capacity of the tumors. The hTid50/hTid48 interaction with APC is involved in the APC-mediated Wg/Wnt function in the morphogenetic processes that signal towards the cytoskeleton and cell polarity. It is observed that any alterations in the functioning of one of the molecular partners consequently lead to changes in the function(s) of the entire complex, and ultimately cause the complete destabilization of these processes. hTid-1 proteins being members of the DnaJ protein family, are essential components of the Hsp70/Hsc70 chaperone machinery and act as an important link between the function of chaperone machines, APC-mediated Wg/Wnt signaling and tumor development [105].

Further, hTid-1 also plays an important role in gliomas of glial tumors as demonstrated in Fig. 5E. The human hTid-1 gene is mapped to the 16p13.3 region of the chromosome, and loss of heterozygosity in this region of the chromosome is an important feature of glial tumors [19]. An interesting study was reported by Trentin et al. [19] with a hTid-1L mutant resulting from a heterozygous frameshift mutation in the SF767 cell line. An anti-apoptotic gain-of-function is exerted in the SF767 cell line, which along with other genetic alterations contributes to the survival and progression of the malignant gliomas. The over-expression of hTid-1L with adenoviral constructs seemed to show no significant effect on the glioma cell growth, however, it was observed that the over-expression of hTid-1S caused significant inhibition of cell proliferation in all the glioma cell lines tested. The ectopic expression of hTid-1S was also found to render the SF767 glioma cell harbouring a mutant hTid-1 allele susceptible to apoptosis while causing growth arrest in wild-type hTid-1-expressing U373 and U87 cells. There had been reports earlier which went on to prove that the susceptibility to detrimental stimuli such as TNFα in O2OS Osteosarcoma cell lines could be reduced via an inducible expression of recombinant hTid-1S [14], which were in sharp contrast to the observations in glioma cells. These observations raise the possibility that hTid-1 splice variants react alternatively to chemosensitization via chemotherapeutic agents, or have alternate effects in different cell types. In a summary, the observations from the different glioma cell lines prove that in gliomas expressing mutated hTid-1, hTid-1S is capable of initiating apoptosis, but not in cells expressing wild-type endogenous hTid-1 proteins [105].

A recent study by Chen et al. [106] also shows that hTid-1 plays an important role in hepatocellular carcinomas (HCC), which comprise more than 80% of primary liver cancer, and is the fourth leading cause of cancer-related deaths worldwide, and ranks second in terms of deaths in men [107, 108]. Chronic infections with hepatitis B virus (HBV) and hepatitis C virus (HCV) are the most common risk factors for HCC [109, 110]. In HCC, sustained Nuclear erythroid 2-related factor 2 (Nrf2) activation, which is a cytosolic transcription factor that acts against oxidative stress, leads to cellular proliferation and resistance to drugs. The commonly followed line of treatments for HCC is surgical resection, radiofrequency ablation (RFA), and liver transplantation. However, the potential issues with these lines of treatments are the high recurrence rate with curative resection and the shortage of organ sources for transplantations [111, 112]. These are the reasons that prompted research into finding a potential biomarker that can help to predict the recurrence of HCC post curative treatment. In HCC tissues, the expression of hTid-1 has been found to be lower as compared to normal liver tissue. In HCC cell lines, the over-expression of hTid-1 was found to inhibit colony formation. Also, patients with higher expression of hTid-1 in the non-tumor part of the liver were found to have higher recurrence-free survival in HCC. Interestingly, it was found that as the tumor progressed, the expression of hTid-1 in the non-tumor part was found to be progressively enhanced. This observation indicates that the expression of hTid-1 in the non-tumor part might reflect an anti-tumor mechanism of humans during tumor progression. Also, lower expression of hTid-1 and higher expression of Nrf2 in the non-tumor part predicted extremely lower chances of recurrence-free survival in the case of HCC. Thus, it can be concluded that hTid-1 plays an important prognostic role in the case of HCC port surgery. Also, the suppression of cancer progression and tumorigenesis by hTid-1 are important events that go on to suggest that hTid-1 can be a promising prognostic marker and potential therapeutic target for HCC [106].

#### Regulating viral oncoproteins

For more than a decade, hTid-1 has been studied for its interactions with viral oncoproteins. In Human Papillomavirus (HPV)-positive cervical cancers, the expression of the E7 open reading frame is consistently conserved [113]. The HPV-16 E7 encodes an acidic phosphoprotein of 98-amino acids and the transforming functions of E7 are related to its ability to interact with host cellular proteins [7]. It is reported that hTid-1 is involved in complex formation with the E7 human papillomavirus oncoprotein, and the cysteine-rich metal-binding carboxyl-terminus domain of E7 is the major determining factor for the interaction. This evidence of the ability of HPV E7 to interact with a cellular DnaJ protein indicates that this viral oncoprotein seemingly targets general regulatory pathways via the J-domains [7].

Another oncogenic viral protein known as Tax is encoded by the human T cell leukemia virus type 1 (HTLV-1) [114], and identification of the cellular binding partners of Tax was extremely important for understanding the molecular basis of Tax-induced cellular transformation. Tax interacts with hTid-1 by its central cysteine-rich domain and induces the cellular transformation of T lymphocytes. Tax interacts and binds to the molecular chaperone complex containing hTid-1 and Hsp70, and causes its sequestration within cytoplasmic “hot spots”, while in the absence of Tax, the expression of the molecular complex of hTid-1/Hsp70 is limited to the perinuclear mitochondrial clusters [16].

Similarly, hTid-1 has also been found to interact with the Epstein Barr Virus (EBV) BARF1 protein and plays important role in the maturation and secretion of the protein by aiding N-Linked glycosylation of the protein on the asparagine 95 residue [115]. The Epstein-Barr Virus (EBV) is a human herpesvirus restricted to B-lymphocytes and epithelial cells. Epstein Barr virus infection is responsible for several human cancers such as Burkitt’s Lymphoma (BL), Nasopharyngeal carcinoma (NPC), and Hodgkin’s Lymphoma. Co-expression studies with hTid1L and BARF1 have shown that hTid1L interacts directly with BARF1, which is in accordance with the yeast two-hybrid screening results. Deletion studies have also shown that two domains in hTid1-1S, viz., 149–320 and 303–453 may also interact with BARF1 [115]. Additionally, the hTid-1 and BARF1 were found to be localized to the perinuclear re58gions of the cell. hTid-1 is generally found to be localized to the mitochondria, however, hTid-1L and BARF1 were not found to co-localize in the mitochondria, rather it was seen that BARF1 could potentially sequester hTid-1L to the endoplasmic reticulum (ER). Contrary to the most researched mitochondrial roles of hTid-1, it was found that a pre-requisite for the physical binding of hTid-1 and BARF1 is the sequestration of hTid1 to the ER and/or the Golgi apparatus [115]. This study is unique in suggesting a mitochondria-independent role of hTid-1. Co-expression of BARF1 with hTid-1 is observed to aid in the secretion of BARF1, thereby suggesting that hTid-1 acts as a chaperone aiding the folding, processing, and maturation of BARF1 [56].

The next important category is Hepatitis B Virus (HBV), a member of the hepadnaviruses, which are DNA viruses replicating through reverse transcription of an RNA intermediate. HBV causes acute and chronic hepatitis and plays a major role in the development of hepatocellular carcinoma (HCC) in humans. Unlike the general role of chaperone proteins, which is to aid viral protein folding, and enhance virus replication, ectopic expression of hTid-1 is found to suppress replication of HBV in transfected human hepatoma cells [116]. hTid1-S was found to directly interact with the viral capsid-like particles of the HBV. In these studies, the interaction with hTid1-L wasn’t studied since hTid1-S is the major form present in the cell. The over-expression studies with hTid-1 were found to accelerate the degradation of the viral core and HBx proteins. On the contrary, the regulation of hTid-1 with RNA-interference was seen to increase viral replication by as much as 83% [116]. These observations prove that cellular chaperones inhibit viral replication via destabilization of viral proteins, and may play suppressive roles in hepatocellular carcinomas.

### Role of hTid-1 in Myogenesis and muscular disorders

#### Myogenesis

Myogenesis is the process in which muscle tissues are formed from the mesodermal layer during embryogenesis [117]. hTid-1 levels crucially affect the normal myogenesis process. During the process of myoblast (C_2_C_12_) differentiation, the protein levels of hTid-1 and the mitochondrial activity were found to be subsequently higher, which indicates that hTid-1 plays an important role during the process of myogenesis [32]. The deficiency of hTid-1 has been found to impair the mitochondrial activity, thereby causing insufficient ATP production and subsequently cellular apoptosis. The mitochondrial biogenesis marker, PGC-_1α_ was also found to be reduced in hTid-1-knockdown C_2_C_12_ cells. hTid-1 deletion in transgenic mice has been shown to cause dysfunction of muscle tissues [32]. These findings indicate that hTid-1 downregulation causes a decrease in ATP production together with increased cellular apoptosis, which is followed by reduced mitochondrial biogenesis. Thereby, the impaired mitochondrial activity of muscle cells during myogenesis consequently causes apoptosis of muscle cells [32]. However, how hTid-1 deficiency reduces ATP production remains to be explored and would be an interesting avenue for further research.

#### Muscular disorders (dilated cardiomyopathy)

Dilated cardiomyopathy (DCM), the most common form of non-ischemic cardiomyopathy is characterized by ventricular chamber dilation and myocyte hypertrophy. Heat-Shock proteins like hTid-1 act as molecular chaperones acting to put in check the aggregation of unfolded polypeptides and help in their proper refolding to form functional peptides. hTid-1 has been seen to exhibit differential expression during developmental stages of cardiac tissue and pathological hypertrophy, and mice deficient in hTid-1/Dnaja3 have been found to have decreased mitochondrial DNA copy number and to develop dilated cardiomyopathy and progressive respiratory chain deficiency, subsequently causing the death of mice before 10 weeks of age. These observations suggest that hTid-1 is essential for mitochondrial biogenesis through its chaperone activity on the α-subunit of DNA polymerase γ (Polga), and plays a necessary role in preventing Dilated Cardiomyopathy (DCM) [31]. The highly expressed Carboxyl-terminus of Hsc70 Interacting Protein (CHIP) shows a strong cardio-protective effect by inhibiting apoptosis following ischemia/reperfusion injury [118, 119]. Hypertensive mice subjects have also been shown to have reduced CHIP and hTid-1 expression. Isoproterenol (ISO) has been found to induce hypertrophy and apoptosis in cardiac myocytes both in vivo and in vitro, by stimulation of the β_1_-adrenergic receptor(β_1_-AR), which signals through a stimulatory G protein (Gs) thereby activating adenylyl cyclase (AC) via its α-subunit (Gαs) and subsequently inducing the formation of cAMP and protein kinase A activation [120]. Similar results were also seen in ISO-induced hypertrophy in H9c2 cell lines, which led to a decrease in the hTid-1 and CHIP expression and subsequent hypertrophy and apoptosis. Over-expression of hTid-1 was seen to cause an increase in endogenous expression of CHIP. Gαs are an important regulator of cardiomyocyte apoptosis and lead to the failure phenotype [121], and it has been found that hTid-1 over-expression caused the degradation of Gαs by CHIP activation, and so targeting Gαs for degradation may have a cardioprotective effect [122]. Lipopolysaccharide (LPS) has been found to induce cellular hypertrophy by upregulation of hypertrophy marker ANP and BNP in cardiomyocytes [123]. Over-expression of hTid-1S has been found to suppress the expression of TLR-4, NFATc3, and BNP proteins, which subsequently caused LPS-induced cardiac hypertrophy inhibition. It has been shown that hTid-1S causes activation of p-PI3K and p ^ser473^ Akt survival protein expression, subsequently leading to inhibition of LPS-induced cardiac hypertrophy, thereby suggesting that hTid-1S causes attenuation of cardiomyoblast cell damages initiated by LPS induction [124].

### Role of hTid-1 in neurodegenerative diseases:

Neurodegenerative diseases are caused by the degeneration and eventual death of nerve cells in the brain or peripheral nervous system, affecting several body activities such as balance, movement, talking, breathing, and heart function. While the role of hTid-1 has been widely studied in different cancer environments, not much was studied about its role in neurodegenerative diseases. Parkinson’s Disease (PD) is a neurodegenerative disease identified by the loss of dopaminergic neurons in the substantia nigra (midbrain dopaminergic nucleus modulating motor movement and reward functions as part of the basal ganglia circuitry) and the a-synuclein (neuronal protein regulating synaptic vesicle trafficking and neurotransmitter release) aggregation into Lewy bodies. The Lewy bodies are abnormal aggregations of proteins that develop inside nerve cells, consisting of alpha-synuclein along with other proteins like ubiquitin, neurofilament protein, and alpha-B Crystallin, occasionally surrounded by neurofibrillary tangles [125, 126]. However, the biochemical pathways in PD which modulate the outcome of protein misfolding and aggregation processes are still not known. Molecular chaperones play important protective roles in protein misfolding diseases like PD by aiding in the proper folding of misfolded proteins [127, 128]. Recent studies show that upon assessment of sensory and motor function and brain region-specific expression levels by western blot analyses in control and PD rats, a 26 kDa breakdown product of the DnaJ fragment of hTid-1 was found in a 6-hydroxydopamine (6-OHDA) PD model of rats, in which gait symmetry, food handling, and sensory performance were found to be compromised [33]. 6-OHDA was used as a PD-mimetic as the 6-OHDA model has been extensively characterized functionally, and the 6-OHDA lesion in the nigrostriatal bundle of rats is comparable to other PD-mimetics because it involves gene transcription changes [129, 130]. The behavioral findings in 6-OHDA rats also mimic the motor and sensory deformities that are typically displayed in human PD patients [33]. The finding of the 26 kDa immunoreactive product of HTID-1, in the PD rat model suggests that hTid-1-mediated stability and proper protein folding are compromised in PD. These results suggested that the changes in cellular levels of hTid-1 caused due to the 26 kDa hTid-1 breakdown product are critical in the pathogenesis of PD hindering the functional and structural compensation and causing an increase in the neurodegenerative processes [33].

Another neurodegenerative disease, Alzheimer’s disease is a neurodegenerative disease affecting a major population of people which causes the death of brain cells and brain atrophy. The deposition of amyloid-beta 42 (Aβ42) is considered to be a very critical factor causing the pathogenesis of AD [131, 132]. Mitochondrial dysfunctions and oxidative stresses play big roles in Alzheimer’s Disease (AD) pathogenesis. Zhou et al. [34], reported that in the brain hippocampal complex of AD patients and Tg2576 mice, upregulation of hTid-1 is observed. Their study shows that in rat cortical neurons, Aβ42 was found to increase the expression of hTid-1, and hTid-1 knockdown prevented Aβ42 induced neuronal cell death. Further, hTid-1 over-expression in HEK293-APP cells increased the BACE1 levels, subsequently augmenting the Aβ levels in the cell. This activates the c-Jun N-terminal kinase (JNK) and amplifies the production of Aβ. These results suggested that hTid-1 induces apoptosis and increases Aβ production in hippocampal brain sections of patients with AD and Tg2576 mice and can hence be studied further for therapeutic intervention for AD [34].

Recently, Patra et al. [133] reported a very interesting case of the first human mitochondrial disease linked to a variant of the hTid-1 protein, c.452G > C (p.(Arg151Thr)), causing intellectual disability, developmental delay, unsteady gait, and peripheral polyneuropathy. This particular variant of hTid-1 is imported into the mitochondria at a lower rate than the wild-type protein, and it was found in a single patient from a consanguineous family. However, the lower import rate of the variant wasn’t necessarily the contributing factor to the deformities observed in the patient. The brain MRI of the patient revealed basal ganglia disease. A key function of hTid-1 is to assist mortalin (human mitochondrial Hsp70) in the reactivation of misfolded or aggregating proteins by accelerating ATP hydrolysis to ADP by using mortalin, thereby enhancing the binding of unfolded proteins to mortalin. hTid-1L was found to induce a fourfold increase in the ATPase activity of mortalin, while the c.452G > C (p. (Arg151Thr)) variant affected the ATPase activity of mortalin by only twofold only. These results show that the hTid-1 c.452G > C (p. (Arg151Thr)) variant functions poorly as a co-chaperone to mortalin as it isn’t able to efficiently regulate the Hsp70 ATPase activity and as a result, the disaggregation function of mortalin-hTid-1 is compromised in the hTid-1 c.452G > C (p. (Arg151Thr)) variant. These effects generate developmental deformities in the patient. This study proves that hTid-1 plays an important role during neuronal development [132].

## Conclusion

The role of hTid-1 is critical to a variety of cellular processes like growth, proliferation, differentiation, senescence, survival, apoptosis, etc., and many studies have been done to have a better understanding of how this particular protein functions in the cell. These studies aimed at understanding how hTid-1 interacts with other proteins in the cell that result in specific responses to cellular and environmental stresses. hTid1 is a DnaJ protein 52 kDa protein that has two common splice variants in humans, i.e., hTid-1L and hTid-1S. The two splice variants have been found to show opposite effects in several cancers and regulation of different proteins in the cell. hTid1 also plays an important role in different signaling pathways of the cell and the anomalies in cellular signaling cascades result in different kinds of cancers and other disease states. hTid-1 plays an important role in eliciting specific responses to these abnormal states in the cell. This review aims at establishing the relations between hTid-1 and its various interacting cellular and viral proteins that play an important role in their specific cellular localizations and responses to different disease states. These observations can be considered monumental in identifying hTid-1 as an important focal point for the development of future therapeutic approaches that could help in designing treatment approaches to complex diseases such as different cancers, dilated cardiomyopathies, and neurodegenerative diseases. The current knowledge about the role of hTid-1 in different diseases can be collaborated with the knowledge of hTid-1 interacting proteins to possibly find out more signaling pathways and their anomalies that result in these diseases. Such holistic information and knowledge about more than one signaling pathway that results in diseases can only help in designing better and more stringent therapeutics to address these complex disorders.

hTid1 plays important roles in several aspects of the cell. hTid1 is an important co-chaperone in the Jak kinase pathway [15]. Co-chaperones play important roles in the cell in assisting the chaperones in substrate protein selection and in aiding the proper folding of misfolded proteins [134]. hTid-1 through its role in the Jak-STAT pathway helps in the control of leukemia [15]. Leukemia is the most common type of cancer in children [135] and Jak kinases are implicated in the pathogenesis of leukemia. Studies by Sarkar et al. [15] show that hTid-1 acts as an important negative regulator of the JAK-STAT pathway. hTid-1 acts as a co-chaperone and causes conformational changes in Hsp70/Hsc70 which cause it to interact with Jak and lead to its inhibition. While the role of co-chaperones has been studied widely in the context of protein misfolding and neurodegenerative disorders, this is an important study that helps to highlight how co-chaperones help in the prevention of disease progression in cancers.

Another important aspect of the role of hTid-1 in the cell is in the proteasomal degradation of proteins which is important in the regulation of gene expression and responses to stresses [136]. It is important to note that hTid-1 plays a pivotal role in the regulation of expression of several proteins that play important roles in cancers, notably, breast and lung cancers. The degradation of ErbB2 and EGFR that play direct roles in the progression of breast and lung cancers are degraded by hTid-1 via the proteasomal pathway which is an important indicator as to why hTid-1 can act as a potential molecule of interest in the future to treat cancers. Additionally, in cancer cells, autophagy suppresses tumorigenicity by inhibition of cancer-cell survival and inducing cell death [137]. Since hTid-1 plays a vital role in autophagy by facilitating the formation of the LC3_autophagosome foci [41], so, it would be interesting to know more about the roles of hTid-1 in autophagy and the cellular pathways by which hTid-1 may affect the process of carcinogenesis via autophagic pathways. hTid-1 not only affects autophagy in cancer but it also plays major role in other diseases like neurodegenerative disorders. In Alzheimer’s disease, the expression of Beclin-1 which is a key component in the formation of the LC3_autophagosome foci is found to be reduced. This leads to the impairment of autophagy in the AD brain which affects mitochondrial clearance and APP processing [138]. Since hTid-1 directly interacts with Beclin-1 and executes autophagy induction, so, this can open up a new arena of research on initiating autophagy in the AD brain which could reduce the accumulation of amyloid-β, and hence improve the disease pathology of AD.

hTid-1 plays important roles in several important signaling pathways of the cell and helps to regulate important cellular processes. APC plays an important role in the Wnt signaling pathway, and hTid-1 is involved in complex formation with APC which helps to regulate cellular proliferation [68]. This is important to ensure the controlled growth and proliferation of the cells, and hence hTid-1 plays the role of a tumor suppressor along with APC. Similarly, hTid-1 interacts with the Trk receptor kinases and helps to regulate synaptic strength and plasticity of the mammalian nervous system [24, 54]. Another important role of hTid-1 in the regulation of synaptic strength is via its interaction with MuSK which helps in proper synaptic transmission [73].

hTid-1 plays an important role in the regulation of two processes i.e., maintenance of the integrity of mitochondrial DNA (mtDNA) and maintenance of homogenous distribution of mitochondrial membrane potential. Loss of mitochondrial membrane potential is a signal for bioenergetic stress and may result in cell death via the release of apoptotic proteins [139]. Since hTid-1 plays an important role in the maintenance of mitochondrial membrane potential, so it would be interesting to also study cellular death via regulation of hTid-1 expression in cancer cells [80]. It might open up new avenues in the future to cause the death of cells whose cellular machinery has gone haywire because of different stresses and cellular insults. Studies by Ng AC et al. [80] show that hTid-1 also plays an important role in the process of myogenesis, as the deficiency of hTid-1 has been seen to cause the impairment of mitochondrial activity and hence death of myoblast cells. This study is fundamental in recognizing that hTid-1 not only plays an important role in several disease states but also is instrumental in the regulation of processes in a healthy body.

On one hand, hTid-1 plays an important role in the process of myogenesis, and on the other hand, hTid1 plays an important role in muscular disorders such as dilated cardiomyopathy. Lower expression of hTid1 has been found to decrease the mitochondrial copy number in cardiac cells and causes dilated cardiomyopathy in mice. hTid-1 has been shown to exhibit a cardio-protective role by degradation of Gαs via increasing the expression of CHIP. Gαs is an important member of the G-protein (guanine nucleotide-binding protein)–coupled receptors (GPCR) family of proteins that play pivotal roles in the physiological regulation of cardiac function and hence can be drug targets for the treatment of cardiac malfunctions [140]. Thus, the interaction of hTid-1 with Gαs can be monumental in identifying hTid-1 as a therapeutic target for cardiac disorders.

While hTid-1 predominantly acts as a tumor suppressor in the cell, there are certain instances, like those in Lung cancers wherein hTid-1 expression results in poor prognosis in different disease phenotypes. The cellular microenvironment and the cellular localization of hTid-1 are also important factors in determining the specific response of hTid-1 in different disease states. The two splice variants of hTid-1, i.e., hTid-1L and hTid-1S localize to different areas in the cell and that causes them to interact with a different array of proteins, thereby eliciting different responses to different cellular and environmental stresses and stimuli. Also, the fact that the two hTid-1 splice variants generally elicit opposing reactions to different cellular processes such as apoptosis is an important determinant of the role of hTid-1 in different diseases and disorders of the cell. It is of importance to note here how Tid1 splice variants affect the fate of Lung cancers differently, as Tid1S has been found to aid in the cancer development of NSCLC patients by enhancing the migration and invasiveness of the malignant cells [84]. On the contrary, higher expression of Tid1L has been found to play a key role in the inhibition of lung adenocarcinomas [61]. However, Tid1L and Tid1S have been shown to affect the gliomas in opposite ways, as higher expression of Tid1S has been seen to affect the survival rate in a more positive manner [107]. In the few neurodegenerative diseases that have been studied in the context of Tid1 Expression, higher Tid1 expression has been found to affect the survival rate of Alzheimer’s disease poorly, while an opposite effect was seen in the case of Parkinson’s Disease.

To have more clarity on the splice variants of hTid-1 and their specific roles, it would be interesting to find out the effect the different splice variants of hTid-1 have on the different disorders that result from hTid-1 expression imbalances. In a summary, hTid-1 lies at the focal point of several important signaling pathways and other processes in the cell, and more research into its altered expression levels in different diseases states and other anomalies is required to develop it into a potential target for varied therapeutic approaches.

## Future perspectives

Owing to the important role that hTid-1 plays in different cancers, cardiovascular diseases, and neurodegenerative disorders, it is evident that more research needs to be done to understand the specific roles of hTid-1 in different diseases and if hTid-1 can be used as a biomarker for the specified human disorders. It has been seen that while the expression levels of most Hsp40 proteins are low in cancer cells and that mostly Hsp40 proteins have been seen to exhibit tumor-suppressive functions, however, in Cancer Stem Cells (CSCs) isolated from a population of Renal Cell Carcinoma (RCC), DNAJB8 expression level was found to be higher, which was suggestive of its role in cancer initiation. A mouse control of RCC vaccinated against DNAJB8 was found to have a significant reduction in tumor size and growth [141] In similar ways, vaccines can be developed against the specific variants of hTid-1 that have been found to show oncogenic functions and aid in tumor development. Even in the case of Alzheimer’s disease, wherein hTid-1 has been shown to increase the levels of Aβ which cause the formation of senile plaques in the brain, vaccines targeting the specific variant of hTid-1 can help in alleviating some of the risk factors of AD. Further research has to be done to shed light on which specific splice variants of hTid-1 cause the disease states. Small molecule derivatives of phenoxy-N-arylacetamides have been found to be effective in inhibiting several Hsp40 proteins [142], and it remains to be seen in the future how effective they are against hTid-1. Thus, to date, it remains to be researched as to whether it would add more value to the life of patients by aiding or inhibiting the expression of hTid-1 in the cell. Depending on the information from these important scientific developments that have helped to more or less understand the role of hTid-1 in different cancers and neurodegenerative disorders and other disorders of the heart, it would add immense value to the life expectancy of patients suffering from these disorders to design specific molecules that can alter the responses caused by the erroneous expression of hTid-1.

## Data Availability

Not applicable.

## Abbreviations

hTid-1: Human-tumorous imaginal disc 1
DNAJA3: DnaJ homolog subfamily A member 3
Hsp: Heat shock protein
HEK293EBNA: HEK 293 cell line expressing the Epstein–Barr virus nuclear antigen-1
Hsc 70: Heat shock cognate 70
STAT: Signal transducer and activator of transcription
mtDNA: Mitochondrial DNA
LC3: Microtubule-associated protein light chain 3
GATE-16: Golgi-associated ATPase enhancer of 16 KDa
GABARAP: G-amino butyric acid type A receptor-associated protein
Wnt: Wingless and Int-1
Trk: Tyrosine receptor kinase
HPV: Human papillomavirus
HTLV-1: Human T cell leukemia virus type 1
Tax: Transactivator from the X-gene region
EBV: Epstein Barr virus
BARF1: BamH1-A rightward frame-1
BL: Burkitt’s lymphoma
NPC: Nasopharyngeal carcinoma
HBV: Hepatitis B virus
HCC: Hepatocellular carcinoma
Jak: Janus kinases
ErbB2: Erb-B2 receptor tyrosine kinase 2
EGFR: Epidermal growth factor receptor
NF-κB: Nuclear factor kappa-light-chain-enhancer of activated B cells
HIF-1α: Hypoxia-inducible factor 1-alpha
pVHL: Von Hippel–Lindau protein
VEGF: Vascular endothelial growth factor
MetR: C-Met receptor tyrosine kinase
HGF: Hepatocyte growth factor
CHIP: Carboxyl terminus of heat shock cognate 70 interacting protein
NGF: Nerve growth factor
MuSK: Muscle-specific kinase
AChR: Acetylcholine receptor
NMR: Neuro-muscular junctions
NDUFS3: NADH dehydrogenase (ubiquinone) iron-sulfur protein 3
NDUFA9-NADH: Ubiquinone oxidoreductase subunit A9
NSCLC: Non-small cell lung cancer
IL8: Interleukin-8
HNSCC: Head and neck squamous cell carcinomas
NCMT: Non-cancerous matched tissues
hnRNP: Heterogeneous nuclear ribonucleoproteins
APC: Adenomatous polyposis coli
TCF/LEF: T-cell factor/lymphoid enhancer factor
TNFα: Tumor necrosis factor-alpha
DCM: Dilated cardiomyopathy
ISO: Isoproterenol
TRPC6: Transient receptor protein channels C6
PD: Parkinson’s disease
AD: Alzheimer’s disease
6-OHDA: 6-Hydroxydopamine
Aβ42: Amyloid-beta 42
BACE-1: Beta-secretase 1
CSC: Cancer stem cells
